# The Role of the Intravenous IgA and IgM-Enriched Immunoglobulin Preparation in the Treatment of Sepsis and Septic Shock

**DOI:** 10.3390/jcm12144645

**Published:** 2023-07-12

**Authors:** Giorgio Berlot, Silvia Zanchi, Edoardo Moro, Ariella Tomasini, Mattia Bixio

**Affiliations:** 1 Azienda Sanitaria Universitaria Giuliano Isontina, Department of Anesthesia and Intensive Care, 34148 Trieste, Italy; zanchi.sil1@gmail.com (S.Z.); edoardo.moro@gmail.com (E.M.); ariella.tpmasini9@gmail.com (A.T.); 2 UCO Anestesia Rianimazione e Terapia Antalgica, Azienda Sanitaria Universitaria Giuliano Isontina, Strada di Fiume 447, 34149 Trieste, Italy; 3 Ospedale Policlinico San Martino, Department of Anesthesia and Intensive Care, 16132 Genova, Italy; mattia.bixio@gmail.com

**Keywords:** sepsis, septic shock, immunotherapy

## Abstract

Polyclonal Intravenous Immunoglobulins (IvIg) are often administered to critically ill patients more as an act of faith than on the basis of relevant clinical studies. This particularly applies to the treatment of sepsis and septic shock because the current guidelines recommend against their use despite many investigations that have demonstrated their beneficial effects in different subsets of patients. The biology, mechanisms of action, and clinical experience related to the administration of IvIg are reviewed, which aim to give a more in-depth understanding of their properties in order to clarify their possible indications in sepsis and septic shock patients.

## 1. Introduction

Although not all their indications are based on studies fulfilling the evidence-based medicine (EBM) criteria, intravenous immunoglobulins (IvIgs) are currently used in a number of diseases in Intensive Care Unit (ICU) patients. These clinical conditions include (a) autoimmune reactions directed against some tissue target(s), such as Myasthenia Gravis, Guillain-Barre Syndrome, etc.; or (b) a systemic response to an infection, including sepsis and septic shock [1].

The rationale for their administration derives from their biological properties, consisting of both the down-regulation of an exaggerated immune response and the enhancement of the immunological capabilities. As far as the use of IvIg in septic and septic shock patients is concerned, ICU physicians can be subdivided into non-believers, who do not use these preparations due to the lack of robust studies fulfilling the strict EBM rules and believers, who instead use them on the basis of a number of investigations and meta-analyses (MAs) demonstrating a positive effect on the outcome.

The aim of this review is to provide a detailed overview of the possible role of IvIg in critically ill septic patients.

## 2. Structures and Function of Immunoglobulins

**Figure 1:**
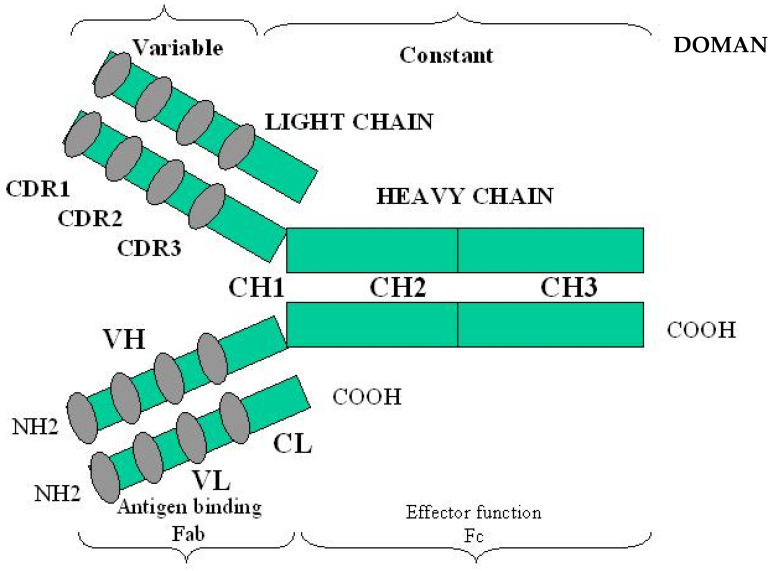
Two-dimensional structure of an IgG molecule. VH and VL indicate the variable regions of the heavy and light chains, respectively. The variable regions located on both the light and heavy chains recognize the epitopes (Fab region). The hypervariable segments located in the Fab regions, which are separated from each other by relatively constant polypeptide chains, are denominated CDR (Complementary Determining Region) domains. The Fc region binds to complement and to the receptors located on the surface of the reticulo-endothelial cells triggering their activation. The region connecting the two functional parts can undergo conformational changes in order to re-shape the molecule according to antigen variability [1].

The immune system works via two different but cooperating arms [2] whose activation is triggered by the adhesion of foreign substances on the receptors located on the surface of the cells involved in the response. The innate system is based on the cells of the reticulo-endothelial complex, the wide number of mediators produced by these cells during the interaction between the host and the invading organisms and the complement cascade. Yet, as the number of the receptors is genetically determined and not implementable, the innate arm cannot cope with the almost infinite variety of microbial epitopes and acts as a first responder, aiming to circumscribe the infection and impede its spread. The second arm, known as adaptative immunity, is more flexible and is based on the production of the Ig, which is encoded by genes that are able to undergo somatic recombination and hypermutations in order to face the myriad of substances coming in contact with the host. The Ig are secreted by plasma cells, which are derived from B lymphocytes that are activated by trapping antigens on a cell-surface receptor and stimulation with CD4^+^ T lymphocytes. Antibodies belong to five different classes of Ig (G, A, M, E, and D, respectively) (Figure 1) [3].

The molecules belonging to the IgG class are considered the prototypical structure and consist of a Y-shaped molecule formed by two identical heavy (H) and light (L) peptide chains. Both chains are divided into a variable (V) domain that reacts with the antigen, and a constant (C) region that activates the various components of the innate immune system, triggering its response (for example, phagocytosis, antibody-mediated and cell-mediated cytotoxicity, and complement-mediated lysis) (Figure 1). The V regions contain three hypervariable regions that are ultimately responsible for the specific shape of each molecule of Ig. Electrostatic forces in association with disulphuric bridges link the H and L regions together.

Therefore, Ig can be considered biochemical transducers able to exert many different actions (Table 1).

For the believers, the different effects exerted by the native Ig on both arms of the immune system could justify the use of IvIgs in clinical circumstances characterized either by (a) an immunodepression due to different causes making the patient prone to newly acquired infections or to the reactivation of latent germs and viruses (see later); or (b) an exaggerated inflammatory response and/or the production of autoantibodies directed against the host’s own tissues, including Guillain-Barrè syndrome, Myasthenia Gravis, thrombotic thrombocytopenic purpura (TTP), etc. As far as the former point is concerned, it should be remarked that, besides other causes of immunodepression, this clinical profile is increasingly recognized in chronic critically ill patients, which are often elderly with a number of comorbidities and frailties who survive the initial insult (i.e., septic shock due peritonitis caused by a colon perforation, pneumonia, etc.) but fail to recover and succumb many days if not weeks after the admission [4,5,6,7,8]. This latter condition is characterized by persistent low-grade inflammation, causing muscle wasting that prevents the weaning from mechanical ventilation, which is associated with an overall down-regulation of the immune capabilities with the subsequent occurrence of ICU-acquired infections (see later).

## 3. The Case of IgM and IgA

Then, once the different actions of the native Ig molecules have been stated, it is worthwhile to describe with more detail the biological properties of the IgM and of IgA. The former is the first Ig to be produced during an infection and has been found throughout all classes of vertebrates, and is present in a dimeric form on the surface of B lymphocytes and circulates as a pentamer (occasionally as a hexamer) in the blood. IgM molecules are the first antibodies produced during infection and appear first during ontogenesis; IgM molecules. Due to their unique structure, IgM molecules form strong interactions with different ligands and have a higher affinity for the complement as compared with IgG [9]. Experimentally, IgMs allow the clearance of apoptotic cells of the reticulo-endothelial system and of the peritoneal macrophages [4]. On the basis of these observations, it is likely that the circulating pentameric IgM molecules bind ligands more avidly than those present on the surface of the B-cells surface but it is not known if, in the presence of reduced blood IgM concentrations, their role could be replaced by the latter [9]. Some investigators demonstrated that IgM concentrations are decreased in septic shock patients and particularly in those with a poor prognosis [10,11]; actually, it appears that reduced levels of this molecule when combined with diminished numbers of natural killer cells (<58 mg/dL and 140 cell/mL, respectively) are associated with an increased risk of death also in non-septic critically ill patients. Should these findings be confirmed in other studies, the supplementation of IgM could be indicated in life-threatening conditions other than sepsis [12].

The IgA molecules are present both in the serum, where they represent the second most prevalent circulating Ig after the IgG, and in the secretions covering the mucosal surface lining the respiratory and the digestive tract, with an overall surface of ~400 m^2^ in the adult. These antibodies are present in two different subclasses, named IgA1 and IgA2 [13]. The IgA molecules exist in multiple forms: in human serum, the prevalent form is monomeric with a subclass distribution of 90% IgA1 and the remaining 10% of IgA2; conversely, in mucosal surfaces, the dimeric form prevails with a more balanced distribution of the two subclasses (40% IgA1 and 60 IgA2). The IgA molecules block or neutralized a number of toxins, bacteria, and viruses and prevent their attachment to the hosts’ cells. In contrast to IgG, IgA does not activate the classical complement pathway and likely activates the alternate one via the lectin pathway.

Overall, it appears that the combined administration of IgA and IgM is valuable as it takes advantage of their dual effect in the bloodstream and in the mucosae.

## 4. Discussion

The history of intensive care medicine is characterized by several hotly debated issues, including the colloid-crystalloids controversy (actually recently replaced by discussions about the best crystalloid solution available), the use of steroids, the appropriate levels of oxygen delivery in critically ill patients, the selective decontamination of the digestive tract, etc. The very same considerations apply to the use of IvIg in septic shock patients and especially for those preparations enriched with supra-normal concentrations of IgM and IgA (eIg). Actually, despite a number of MAs and systematic reviews (SRs) that have demonstrated their efficacy, the current guidelines of the Surviving Sepsis Campaign (SSC) [14] strongly discourage their use primarily due to the absence of large studies robust enough to fulfill the EBM criteria. This notwithstanding, these preparations are widely used in septic shock patients as an add-on treatment aiming not to replace antibiotics or surgery but to enhance the immune capabilities.

To better define the possible role of IvIg and eIg and their possible rules of engagement (ROE) in the treatment of septic shock, it is worthwhile to split the description in different sections.

### 4.1. Why to Give eIg?

According to the current definition, sepsis and septic shock are life-threatening conditions caused by a dysregulated host’s response to an infection leading to multiple organ dysfunctions [15]. Patients with sepsis often present multiple features of immunological alterations, including an initial hyperinflammatory condition that can be followed later on by immunosuppressive events, complement consumption, defects in neutrophil-mediated immunity, and decreased serum levels of immunoglobulins [12]. Sepsis is initiated by the activation of multiple signaling pathways following the recognition and specific cell-surface receptors on cells (toll-like receptors) of pathogen-associated molecular patterns or damage-associated molecular patterns (PAMPs and DAMPs, respectively); the subsequent step consists of the activation of genes involved in inflammation, adaptive immunity, and cellular metabolism, that ultimately determine to the production and release of a huge array of mediators with either pro- and anti-inflammatory capabilities whose respective concentrations vary according to the different phases of the clinical course [5,8].

A number of investigations demonstrated that in septic shock patients, the levels of IgG, IgA, and IgM were decreased, albeit with different effects on the outcome [16,17,18,19,20,21], and that their contemporaneal reduction was associated with reduced survival [22,23,24]. Different mechanisms acting alone or in combination can account for these findings, including (a) the reduced secretion of Ig; (b) their leakage into the interstitial space due to the endothelial dysfunction; (c) their redistribution into the inflamed tissues; and (d) their consumption by the complement system [16,21,22,25,26]. However, in a more advanced phase of sepsis, independently from the initial trigger(s), the hyperinflammatory response subsides and in several cases is replaced by a down-regulation of the immune capabilities. Different factors account for these findings, including (a) the increase in the circulating levels of myeloid-derived suppressor cells (MDSCs) that secrete multiple anti-inflammatory cytokines, such as IL-10 and transforming growth factor-β (TGF-β), which blunts the immune function [27,28]; (b) the reduction in committed antigen-presenting dendritic cells and monocytes with the subsequent loss or severe reduction in the associated production of proinflammatory cytokine [29,30,31,32,33]; (c) the depletion of human leukocyte-antigen D related (HLA-DR) in monocytes and dendritic cells also decreases, with the subsequent reduction in responsiveness [34]; (d) the depletion of circulating lymphocytes along with an increase in the apoptosis of dendritic, l cells, T- and B-cells [8]; (e) the upregulation of the immunosuppressant molecules programmed death protein 1 (PD-1), the programmed death ligand 1 (PD-L1) in monocytes and T lymphocytes that eventually determine (a) the expansion of the regulatory T (T-reg) and unresponsive T-cells [28,35,36]; and (b) a down regulation of both the adaptive and innate immune responses [37,38,39]. The clinical consequence induced by these mechanisms is a persisting low-grade inflammatory state accompanied by an unrelenting hypercatabolism with subsequent muscle wasting and difficult weaning from the mechanical ventilation, the occurrence of re-infections with low-virulence germs, such as Acinetobacter baumanii and the reactivation of viral strains, including Cytomegalovirus and different Herpesviridae.

### 4.2. Which Are the Available Preparation?

As stated above, the available IvIg preparations can be subdivided into those containing the different classes of immunoglobulins roughly at or slightly above their plasmatic levels and eIg and those containing increased concentrations of IgM and IgA (Table 2).

Presently, standard IvIgs and Pentaglobin^®^ have been used in the treatment of septic shock from multiple causes, whereas Triglobin^®^ has been used in an RCT involving septic patients with severe community-acquired pneumonia [40].

Although all polyclonal IvIgs share similar effects on the inflammatory and immune mechanisms and represent a valuable approach to modulate both the pro- and anti-inflammatory processes, differences exist among the various available preparations. Actually, while different studies demonstrated that the administration of IvIg preparations containing only IgG was not associated with improved mortality rates in patients with sepsis [41,42,43], some MAs and SRs have concluded that patients given eIg presented a reduction in mortality of up to 18% [44,45,46,47].

### 4.3. Who Are the Best Candidates for eIg (and Who Are Not)?

As stated above, it appears that possible candidates for eIg can be basically subdivided into two groups according to their clinical features (Table 3).

The former includes patients at the onset of septic shock who can take advantage both from the antibacterial properties of eIg and of their modulation of the early hyperinflammatory response, whereas the latter includes those with an immunocompromised phenotype, often with a prolonged length-of-stay in the ICU who develop a chronic critical illness whose features have been described in the preceding paragraphs. Due to the unrelenting aging of the population in Western countries, it is likely that in the next few years, the number of these patients will increase [48]. As far as the responsible germs are concerned, septic shock patients due to Gram-negative infections are most likely to take advantage of eIg; among Gram-positive strains, a positive effect on the outcome has been reported in patients with severe invasive group A streptococci infections, especially in streptococcal toxic shock syndrome associated with myositis or fasciitis [49]. As far as either the site of infection or non-responding patients are concerned, it appears that (a) the eIg have been successfully used independently from the initial inoculum; but (b) in different studies, patients with hematological diseases did not benefit from their administration [50].

### 4.4. When to Treat?

The SSC guidelines consider sepsis and septic shock as time-dependent clinical entities [14]. This assumption is based on different investigations that demonstrated an association between each hour of delay in the administration of (appropriate) antibiotics and a measurable increase in the mortality rate [51,52,53]. That said, due to the absence of clinical evidence, the most appropriate window of opportunity for the administration of eIg remains substantially undetermined. Consequently, it appears that different approaches can be used (Table 4).

The first is based on the measurement of circulating concentrations of the different classes of Ig that are frequently reduced in septic shock patients; actually, as stated above, several if not all investigations demonstrated an association between low blood levels of native Ig and the outcome of septic patients. This issue appears somewhat controversial because, whereas some investigators found that either isolated or combined low levels of IgG, IgM, and IgA were associated with a decreased survival [16,17,18,19,20] and Giomarellos-Bourboulis et al. [11] showed that the transition from severe sepsis to septic shock and death was marked by decreased blood levels of IgM, other authors reported different results; actually, in a recent meta-analysis (MA), Shankar-Hari et al. [21] demonstrated that low levels of IgG and IgM in septic patients were not associated with a poor outcome. Then, whereas it appears reasonable to restore abnormally low levels of Ig, it is not clear (yet) whether “normal” levels can be considered appropriate in septic shock as their consumption is likely increased as compared to non-septic conditions [54].

Another potential method of guiding the administration of IgM-enriched immunoglobulins is the use of a scoring system, such as the Torino (TO)-PIRO score [55]. This has been developed on the basis of multiple investigations and differs from the original PIRO system [56] as it does not describe the clinical course but rather the underlying medical conditions favoring the occurrence of septic shock, such as the underlying chronic disorders (predisposition), the possible precipitating factors (insult), the host’s reaction to the infection (response), and the possible systemic complications (organ) (Table 5).

Then, on the basis of the score, it could be possible to identify patients who could benefit from the administration of eIg (Table 6).

The last approach basically consists of the administration of eIg immediately after the diagnosis of septic shock. Berlot et al. [57] demonstrated that in 355 septic shock patients, there was an increase of ~2% mortality rate for each 24 h delay in the administration of eIg; however, as stated by the authors, this approach is flawed, which is an inherent risk of approximation as the onset of septic shock is not always immediately recognizable especially when it occurs outside the ICU. However, the score has its own inherent limitations and requires validation in clinical practice and using results gathered from large databases.

### 4.5. Which Dosage?

The summary of product characteristics currently recommends eIg therapy at a dose of 0.25 g/kg body weight/day for 3 consecutive days, but further infusions may be required according to the clinical course. Actually, even if the appropriate levels of immunoglobulins are far from being established, it is reasonable to increase their levels rapidly. To this aim, Rodriguez et al. [55] used a higher dose of eIg (1–2 mg/kg/day for 5 days) in a group of severe sepsis and septic shock surgical patients and observed an improved outcome in the treatment as compared to the control group. Moreover, in order to adapt the treatment to the patients and not vice versa, two different RCTs are currently underway; in the former, the dose of eIg is titrated on the levels of native IgM [58], and in the latter, on the concentrations of some immunologic biomarkers [59]. In order to achieve a patient-tailored treatment, it could be useful to perform repeated measurements during the eIg infusion to measure the circulating levels of the different classes of Ig in order to make sure the dose is adequate according to their and/or variations. Recently, Berlot et al. [54] demonstrated in a group of septic shock patients that the trajectories of IgM and IgA differed between survivors and non-survivors since, whereas the IgG and the IgA increased in both groups, in survivors, the IgM more than doubled at the end of the infusion and almost tripled 7 days later. Different mechanisms can account for these findings, including (a) the ongoing production of endogenous IgG and IgA possibly associated with the reduced production and/or the consumption of IgM in non-survivors; (b) a higher pathogen or PAMP load and the consequent increased opsonization and clearance of the IgM molecule; and (c) the leaking from the bloodstream into the interstitial space of IgM through a more permeable capillary endothelium of non-survivors.

## 5. Original Sins & Open Issues

The skepticism of the SSC guidelines concerning the use of eIg stems from the original sins of the published studies that prevent definite conclusions from being drawn from these studies. The main limitations of these investigations include:

(a) The uncertainness of the timing of administration in relationship with the onset of septic shock appears to be a relevant issue as the outcome of these patients appears to be a time-dependent variable.
(b) The not always indicated appropriateness of the concomitant treatments, such as antibiotics and surgical drainage of septic foci.
(c) The lack of risk stratification of patients, which is very often lumped together without taking into consideration the underlying chronic diseases and subsequent frailties.
(d) The clinical phase of their administration; actually, as stated above, the immune conditions of septic patients can vary according to the time elapsed from the initial insult.
(e) The absence of information about the blood concentrations of native Ig, as well as of other immunological variables before the administration of the eIg.

## 6. Frequently Asked Questions

(a) Is it possible to give eIg to patients undergoing renal replacement treatments and/or other forms of blood purification? Yes, because their molecular weight is too high to be removed or absorbed by commonly used devices; however, they can be removed by plasma exchange [60]. In this case. the eIg should be given after the procedure.
(b) What are the possible harmful side effects? The eIg are well tolerated; however, either a hyperviscosity syndrome or acute renal failure have been occasionally reported, which were likely due to the stabilizers rather than the Ig molecules [61]. Both occurrences can be prevented by adequate hydration.

## 7. Conclusions

Independently from their composition, the use of IvIgs in sepsis is a widespread practice not encouraged by the current SSC as the published studies are flawed by a number of biases, including the heterogeneity of the enrolled patients, the different ROE, the often unspecified timing of initiation, the appropriateness of the antibiotic treatments, etc. [62]. Even taking into account these original sins, they are valuable adjunctive measures if administered as soon as possible after the onset of septic shock. A step toward precision medicine could be constituted by the titration of the dose according to the patient’s immunological or biochemical response aiming for a personalized approach rather than a “one-size-fits-all” policy.

## Figures and Tables

**Table 1:** Mechanisms of action of the immunoglobulins. ↑: increase; ↓: decrease.

Mechanism	Aims
Toxin inactivation	↑ Clearance of endotoxin and exotoxins ↓ Bacterial cell adherence, invasion, and migration
Stimulation of the leukocytes and serum bactericidal action	↑ Endotoxin-induced neutrophil oxidative burst (7S-IvIgG) ↓ Endotoxin-induced neutrophil oxidative burst (5S-IvIgG; F(ab′)2 fragments and IgM) Enhancement of serum opsonic activity
Modulation of cytokine effect	↓ Pro-Inflammatory mediators ↑ Anti-Inflammatory mediators Cytokine neutralization by anti-cytokine antibodies

**Table 2:** Ig concentration in different preparations. * Not yet available.

Variable		Standard Preparations		IgM and IgA-Enriched Preparations	
Ig Class (%)	Normal Plasma Values	Privigen^®^ CLS Behring, Bern, Switzerland	Polyglobin^®^ Bayer Biol. Prod., Leverkusen, Germany	Pentaglobin ^®^ Biotest, Dreiech, Germany	Triglobin^®^ * Biotest, Dreiech, Germany
Ig G	80	>98	>97	56	76
Ig M	7	Traces	Traces	12	23
Ig A	13	Traces	Traces	12	21
Clinical experiences in ICU patients		Septic shock Autoimmune disorders	Septic shock Autoimmune disorders	Septic shock	Severe Community-Acquired Pneumonia

**Table 3:** Different clinical courses of septic patients. Legend: ↑: Increase; ↓: Decrease; ↑↑: Marked increase.

Variable	Early Response	Late Response
Patient population	Young, middle-aged	Elderly
Comorbidities	Often absent, no or few frailties	Present, often multiples
Microorganisms	Highly virulent, toxin releasing	Low virulence, opportunistic Viral reactivation
Clinical phenotype	Septic shock, high fever, ARDS, fast-evolving MODS, community-acquired infections	Altered mental status
Laboratory findings	↑↑/↓ White blood cell ↑ Lactate levels	↓ Lymphocytes
Possible clinical trajectories	Resolution of sepsis Restoration of the immunitary capabilities Early deaths	Protracted ICU length of stay Hypercatabolism and protein waste Difficult weaning from the mechanical ventilation Late deaths

Legend: ARDS: acute respiratory distress syndrome; MODS: multiple-organ dysfunction syndrome.

**Table 4:** Possible criteria for the initiation of eIg.

Trigger	Pros	Cons
Low circulating Ig levels	Physiological basis	Long turnaround time not available on a H24-basis throughout the week; Unknown appropriate levels of IgA and IgM in septic shock
Prediction of septic shock (TO-PIRO)	Easy to assess	None
Time lag since the onset of septic shock	Easy to assess	Time lag often approximated

**Table 5:** The TO-PIRO score.

Items	Criteria	Score
Predisposition	Uncontrolled cancer	1
	Colonization with MDR bacteria and/or candida	1
	Neutropenia or immunosuppression or allogenic stem cell transplant or splenectomy	2
Insult	Necrotizing fasciitis, invasive meningococcal or pneumococcal disease, MRSA	5
	MDR infections or nosocomial infections	2
	Secondary or tertiary peritonitis	2
Response	Leukocytes < 600/mL	2
	IgM < 60 mg/dL	2
	PCT > 10 ng/L or CRP > 20 mg/dL	1
	PCT > 100 ng/L–Il-6 > 1000 pg/mL–endotoxin > 06–presepsin > 1400 ng/L	2
	Disseminated intravascular coagulation	1
Organ	Septic shock	3
	Sepsis with >1 organ failure	2
	Infection without sepsis	1

Legend; MDR: multiple-drug resistant; IgM: M-class immunoglobulin; MRSA: methicillin-resistant S. aureus; PCT: procalcitonin; CRP: C-reactive protein.

**Table 6:** Possible approaches according to the score values.

TO-PIRO Score	Suggestions	Timing
<5	The administration of eIg may be beneficial	Undetermined
5–10	The administration of eIg is suggested	Possibly within 24 h of clinical presentation
>10	The administration of eIg is recommended *	As soon as possible and within 6 h

* Evidence showed reduced mortality in septic shock patients treated with eIg as compared with non-treated patients.

## Data Availability

All data are available.
